# Targeting CD133 improves chemotherapeutic efficacy of recurrent pediatric pilocytic astrocytoma following prolonged chemotherapy

**DOI:** 10.1186/s12943-017-0593-z

**Published:** 2017-01-31

**Authors:** Guifa Xi, Yuping Derek Li, Gordan Grahovac, Veena Rajaram, Nitin Wadhwani, Tatiana Pundy, Barbara Mania-Farnell, Charles David James, Tadanori Tomita

**Affiliations:** 1 Falk Brain Tumor Center, Division of Pediatric Neurosurgery, Northwestern University Feinberg School of Medicine, 225 E Chicago Ave, PO Box #28, Chicago, IL 60611 USA; 2 Development Biology Program, Stanley Manne Children’s Research Institute, Ann & Robert H. Lurie Children’s Hospital of Chicago, Northwestern University Feinberg School of Medicine, Chicago, IL USA; 3 Department of Neurological Surgery, Northwestern University Feinberg School of Medicine, Chicago, IL USA; 4 Department of Pathology, Ann & Robert H. Lurie Children’s Hospital of Chicago, Northwestern University Feinberg School of Medicine, Chicago, IL USA; 5 Department of Pathology, Children’s Medical Center, UT Southwestern Medical Center, Dallas, TX USA; 6 Department of Biological Sciences, Purdue University Northwest, Hammond, IN USA

**Keywords:** CD133, MDR1, Chemotherapy, Pediatric pilocytic astrocytoma

## Abstract

**Background:**

Pilocytic astrocytomas (PAs) are the most common pediatric central nervous system neoplasms. In the majority of cases these tumors are benign and receive favorable prognosis following gross total surgical resection. In patients with progressive or symptomatic tumors, aggressive surgical resection is generally not feasible, thus radiation or chemotherapy are accepted initial or adjuvant interventions. Due to serious long-lasting side-effects, radiation is limited in young children; therefore, chemotherapy is widely practiced as an adjuvant treatment for these patients. However, chemotherapy can promote the emergence of multidrug resistant tumor cells that are more malignant than those of the original tumor. CD133, a putative stem cell marker in normal tissue and malignant brain tumors, enhances multidrug resistant gene 1 (MDR1) expression following chemotherapy in adult malignant glioblastomas. This study examines the relationship between CD133 and MDR1 in pediatric PAs exposed to chemotherapy, with the goal of identifying therapeutic targets that manifest as a result of chemotherapy.

**Methods:**

Slides were obtained for 15 recurrent PAs, seven of which had received chemotherapy prior to surgical treatment for the recurrent tumor. These samples, as well as primary tumor tissue slides from the same patients were used to investigate CD133 and MDR1 expression via immunofluorescence. Archived frozen tissue samples from the same patients were used to examine CD133, MDR1 and PI3K-Akt-NF-κB signaling mediators, via western blot. Two drug resistant pediatric PA cell lines Res186 and Res199 were also used to evaluate the role of CD133 on cell response to cytotoxic therapy.

**Results:**

CD133 and MDR1 were co-expressed and their expression was elevated in recurrent PAs from patients that had received chemotherapy, compared to patients that had not received chemotherapy. PI3K-Akt-NF-κB signaling mediator expression was also elevated in recurrent, chemotherapy-treated PA. Suppressing CD133 expression with siCD133 decreased levels of PI3K-Akt-NF-κB signaling mediators and MDR1, while increasing cell chemosensitivity, as indicated by quantification of apoptotic cells following chemotherapy.

**Conclusions:**

CD133 contributes to multidrug resistance by regulating MDR1 levels via the PI3K-Akt-NF-κB signal pathway not only in adult glioblastomas, but also in pediatric PAs. Targeting CD133, adjuvant to conventional chemotherapy may improve outcomes for children with recurrent PA.

**Electronic supplementary material:**

The online version of this article (doi:10.1186/s12943-017-0593-z) contains supplementary material, which is available to authorized users.

## Background

Pilocytic astrocytomas (PAs), frequently seen in children and young adults, are the most common pediatric central nervous system (CNS) neoplasm [1]. These tumors are primarily treated with surgical resection, with gross total resection curative in the majority of cases [2]. However, for those cases that are not surgically curable, patients receive radiation or chemotherapy [2]. Focal radiation with standard doses of 45 to 54Gy are effective in long-term tumor control, but cause serious side-effects, including decreased intellectual function, endocrine deficits, secondary neoplasms, hearing loss and vasculopathy. As a result, the use of radiation in young children is limited [3]. Chemotherapy has been used as a first-line treatment to delay or replace radiotherapy in certain situations, such as critical tumor location, or in relapsed tumors after surgery [4, 5].

Vincristine, carboplatin and combinations of procarbazine, thioguanine and lomustine are often used to treat pediatric PAs that are not surgically curable. These treatments result in 5-year event-free survival rates of 40–50% [6]. A common consequence of chemotherapy is the development of multidrug resistance, with associated tumor relapse and progression. In this scenario, prognosis is very poor [7]. Primary mechanisms for acquisition of drug resistance include overexpression of ATP-binding cassette (ABC) transporters, such as multidrug resistant protein 1 (MDR1, also known as P-glycoprotein or P-gp), or multidrug resistance proteins (MRPs) [7–9], which pump anticancer agents out of the cells. In pediatric low-grade gliomas, including PAs, MDR1 mediated drug resistance is the main mechanism for chemotherapeutic resistance [10, 11]. Presently there is no clinically effective treatment to compensate for the effects of MDR1, even with substantial research addressing this need.

The cell surface marker, CD133, has been identified as a putative stem cell marker in normal and malignant brain tissues. CD133 and MDR1 co-express at high levels after prolonged chemotherapy in pediatric medulloblastomas [12] and ependymomas [11]. However, direct evidence supporting a relationship between CD133 and MDR1 is lacking. We previously identified enriched levels of CD133 positive cells in adult glioblastoma cultures subjected to prolonged chemotherapy, and determined that CD133 regulates MDR1 expression via PI3K/AKT/NF-κB signaling, in these cells [13]. Because CD133 positive cells are present in pediatric low grade gliomas including PAs [14, 15], in the present study we investigated the potential relationship between CD133 and MDR1 in pediatric PAs, with the goal of identifying therapeutic targets for recurrent tumors subsequent to chemotherapy.

## Methods

### Chemicals and reagents

Doxorubicin (Dox, Cat#44583), vinblastine (VIN, Cat#V1377), vincristine (VCR, Cat#V8388) and verapamil (Cat#V4629) were purchased from Sigma-Aldrich (St. Louis, MI, USA) and prepared following manufacturer’s instructions. CD133 expression plasmid pCMV6-CD133-Myc-DDK, its control vector pCMV6-Myc-DDK, and transfection reagent TurboFectin 8.0 were purchased from Origene (Rockville, MD, USA). Human specific CD133 short interfering (si) RNA (oligonucleotide ID# HSS113055), scrambled control siRNA oligonucleotide (12935-200), and siRNA transfection reagent Lipofectamine® RNAiMAX were purchased from Invitrogen (Life Technologies, Grand Island, NY, USA).

### Clinical samples

**Fig. 1:**
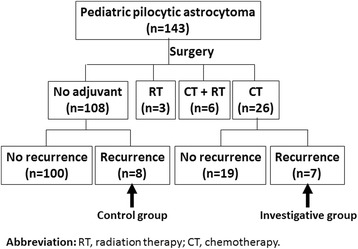
Selection of control and investigative groups

Data for 143 pediatric PA patients was reviewed from the database of the Division of Pediatric Neurosurgery at Ann & Robert H. Lurie Children’s Hospital (A&RLCH) (Additional file 1). Fifteen patients who had primary and recurrent tumor resection were selected for further analysis. Eight of these patients did not receive adjuvant therapy, and are labeled as negative controls; seven received chemotherapy following resection and are labeled as the investigative group (Fig. 1). Detailed clinical characteristics of these 15 patients are presented in Table 1. Survival curves for these 15 patients and hazard ratio were plotted and analyzed using GraphPad Prism 7 (GraphPad Software, Inc. La Jolla, CA USA). Hematoxylin and eosin (H&E) stained sections from formalin fixed paraffin-embedded (FFPE) primary and relapsed tumors samples were independently reviewed by two pediatric pathologists (N.W. and V. R.), using World Health Organization 2007 criteria for tumor classification. Representative slides were immunostained to evaluate MDR1 and CD133 co-expression. The study was approved by the institutional review board (IRB) at A&RLCH.

**Table 1:** Patients characteristics

Patient ID#	Sex	Age (Year)^a^	Site	Relapse time (months)^b^	Chemotherapy/Length(months)^c^	Survival
Control patients						
1	M	5	Spinal Cord	27		Alive
2	M	11	Spinal Cord	30		Deceased
3	M	2	Cerebellum	31		Alive
4	M	11/12	Cerebellum	8		Alive
5	F	4	Left Thalamic	19		Alive
6	M	16	Left intraventricular	58		Alive
7	F	13	Right Front Lobe	20		Alive
8	F	5	Right lateral ventricle subependymal	107		Alive
Investigative patients						
9	M	4	Cerebellum	8	CPL, VIN/12, TPCV/14	Alive
10	M	3	Left Temporal Lobe	13	CPL/4, VCR/4, VIN/10	Alive
11	F	4	Right Temporal Lobe	8	CPL/8, VCR/8	Alive
12	F	7	Spinal Cord	12	CPL/7, VCR/7, ISP/4	Deceased
13	F	1	Suprasellar/Third ventricle	46	CPL, VCR/4, VIN/9, O^6^-BG & TMZ/2	Deceased
14	F	1	C4-T4 spinal cord	27	CPL, VCR, TMZ/12	Alive
15	M	7/12	Posterior Fossa	10	CPL, ETO, TAM/10	Deceased

^a^Age at initial diagnosis

^b^Time of relapse after first operation

^c^Initial chemotherapy/length of treatment (months) after first operation

Abbreviations: *M* male, *F* female, *CPL* carboplatin, *VCR* vincristine, *VIN* vinblastine, *TPCV* Thioguanine, Procarbazine, CCNU and Vincristine, *ISP* ispinesib, *ETO* etoposide, *TAM* tamoxifen, *O* ^*6*^ *-BG* O6-Benzylguanine, *TMZ* temozolomide

### Cell culture and induction of drug resistance

Pediatric PA cell lines Res186 and Res199 were generously provided by Dr. Silber (University of Washington, Seattle, WA, USA) [16, 17]. Cells were grown as monolayers in DMEM/F12 Ham’s medium + 10% FCS in 5% CO2. These cells are labeled as wild-type (WT). Res186 and Res199 cell sublines resistant to doxorubicin (DOX), vinblastine (VIN) or vincristine (VCR) were established as previously described [18]. Briefly, drug resistant cells were obtained by successive exposure to increasing amounts of DOX (0.01 and 0.1 μg/ml), VIN (0.001, 0.01 and 0.1 μg/ml), or VCR (0.001, 0.01 and 0.1 μg/ml). Cells that survived a minimum of five passages at the highest drug dosage were used in this study. These cells were labeled DOX-R, VIN-R and VCR-R and maintained in complete culture medium with indicated drug.

### Immunofluorescence

Formalin-fixed, paraffin-embedded (FFPE) slides for 15 primary PA tumor samples and paired relapsed tumors from eight negative control and seven investigative patients were requested from the Department of Pathology at A&RLCH under IRB protocol#2005-12252. Immunofluorescence followed deparaffinization with 100% xylene and ethanol. Antigen retrieval was performed by boiling for 10 min in 0.01 M sodium citrate (pH 6.0) solution. Endogenous peroxides were blocked with 3% hydrogen peroxide, 10% donkey serum and 0.3% Triton X-100 in PBS. The samples were incubated with rabbit polyclonal MDR1 [EPR10364] (Abcam, ab170904, dilution 1:100) and mouse monoclonal CD133 (Abcam, ab15580-100, dilution 1:100) antibodies, to examine MDR1 and CD133 co-expression. Secondary antibodies were donkey anti-mouse cy3 or anti-rabbit Alexa Fluor 488 (dilution 1:200) (Jackson Lab, ME, USA). Nuclei were counterstained with 4′, 6-diamidino-2-phenylindole (DAPI). Images were captured with light (Leica **DMR-HC** upright microscope), and confocal (Zeiss LSM 510) microscopy and analyzed using OpenLab 5.0 software.

For immunofluorescence of cultured cells, 5 × 10^3^ Res186 and Res199 WT or drug resistant cells were grown on 8-well chamber slides overnight and fixed with 4% paraformaldehyde in PBS (Pierce Chemical Co., Rockford, IL). Fixed cells were blocked with 10% donkey serum and 0.3%Triton X-100 in PBS and incubated with rabbit polyclonal MDR1 [EPR10364] for single staining; or rabbit polyclonal MDR1 [EPR10364] (Abcam, ab170904, 1:100) and mouse monoclonal CD133 antibody (Abcam, ab5558, 1:100) to observe co-expression. Alexa Fluor 488 or cy3 labeled secondary antibodies (dilution 1:200) (Jackson Lab, ME, USA) were used for detection. Nuclei were counterstained with DAPI. Images were captured with a Leica DM-IRB inverted microscope and analyzed using OpenLab 5.0 software.

### MTS assay for determination of cell viability

Cell viability was determined using the 3-(4, 5-dimethylthiazol-2-yl)-5-(3-carboxymethoxyphenyl)-2-(4-sulfophenyl)-2H-tetrazolium (MTS) (Promega) assay with the results read on an ELISA Reader from TECAN Sunrise™ (TECAN, CA, USA). To determine cell viability of Res186 and Res199 WT cells overexpressing CD133 in response to DOX, VIN or VCR, 1 × 10^6^ cells were plated in T25cm^2^ flasks 1 day prior to pCMV6-Myc-DDK or pCMV6-CD133-Myc-DDK transfection using TurboFectin 8.0 following manufactures’ protocol. The cells were harvested after 48 h, plated at 2 × 10^4^ cells/100 μl in 96-well plates with complete medium containing 0.01 μg/ml DOX, VIN or VCR and incubated at 37 °C with 5% CO_2_. After 48 h MTS reagent was added and cell viability was determined following manufactures instructions.

To evaluate cell viability of Res186 and Res199 DOX-R, VIN-R and VCR-R cells treated with 10 μM verapamil (diluted with methanol), drug resistance cells were grown in drug-free media and serum starved for 24 h, then 5 × 10^3^ cells were plated per well in 96-well plates and incubated overnight in complete drug-free media. The medium was then replaced with complete media containing 10 μM verapamil or 10 μl methanol (dilution solvent control) plus 0.1 μg/ml DOX, VIN or VCR. To determine cell viability of drug resistant cells treated with CD133 siRNA, 1 × 10^6^ drug resistant cells were plated in T25cm^2^ flasks 1 day prior to transfection with siCD133 or control siRNA (final concentration 20nM) using Lipofectamine® RNAiMAX following the reverse transfection protocol, as per manufactures instructions. The cells were harvested after 48 h, plated at 2 × 10^4^ cells/100 μl in 96-well plates and incubated overnight in complete drug-free media at 37 °C with 5% CO_2_. The next day culture media was replaced with complete medium containing 0.1 μg/ml DOX, VIN or VCR. After 72 h MTS reagent was added to verapamil or CD133 siRNA treated drug resistant cells and cell viability was determined following manufactures instructions.

Each of the described treatments was repeated three times in triplicate wells. Cell survival is presented as a percentage of viable cells compared to corresponding viable cell number in relevant dilution solvent or control siRNA treatment groups normalized to one hundred percent. Statistical analysis was carried out using GraphPad Prism 7. *P* values were calculated using 2-sided Student’s t test, with *p* < 0.05 considered significant.

### Nuclear fragmentation and flow cytometry for detection of apoptotic cells

To quantify nuclear fragmentation, 1 × 10^4^ WT or drug resistant cells/well were grown in complete or drug-free media on 8-well chamber slides overnight. The following day new media containing 0.01 μg/ml DOX, VIN or VCR for WT cells or 10 μM verapamil plus 0.1 μg/ml DOX, VIN or VCR for drug resistant cells was added to the cells for 72 h. For studies examining the effects of CD133 on nuclear fragmentation, WT cells were transfected with pCMV6-CD133-Myc-DDK or pCMV6-Myc-DDK using Turbofectin 8.0, then placed in complete media containing 0.01 μg/ml DOX, VIN or VCR, for 48 h; drug resistant cells were treated with siCD133 or control siRNA (final concentration 20nM) for 48 h, using Lipofectamine® RNAiMAX, following the reverse transfection protocol as per manufactures instruction, then placed in complete media containing 0.1 μg/ml DOX, VIN or VCR, for 72 h. After drug treatment cells were washed with PBS and fixed with 4% paraformaldehyde in PBS (Pierce Chemical Co., Rockford, IL). Nuclei were stained with DAPI. Images were captured from at least five different fields per well with a Leica DM-IRB inverted microscope and analyzed using OpenLab 5.0 software. Data from a minimum of three independent wells was used to quantify the number of fragmented nuclei, and the results were graphed with GraphPad Prism 7 software. *P* values of less than 0.05 were considered statistically significant.

For flow-cytometry, 5 × 10^6^ cells were grown in 10 cm petri-dishes (BD Falcon) following the same treatment protocol described for quantification of nuclear fragmentation. Floating and attached cells were harvested, fixed and stained with propidium iodide (PI, Life Technologies). Apoptotic cells were determined with sub-G1 flow-cytometric analysis by FACSCalibur flow cytometry (BD Company). Data from a minimum of three independent dishes was used to quantify the number of apoptotic cells and graphed with GraphPad Prism 7 software. *P* values of less than 0.05 were considered statistically significant.

### Quantitative real- time PCR

5 × 10^4^ Res186 and Res199 WT or drug resistant cells were grown in 6-well plates (BD Falcon) and treated following the protocol described for quantification of nuclear fragmentation. Total RNA was isolated with the RNeasy Mini Kit (Qiagen, Valencia, CA, USA). cDNA was synthesized with qScript cDNA SuperMix (5 ×) (Quanta Biosciences, 95048-025) following real-time (RT) PCR with human ABCB1 forward 5′-cccatcattgcaatagcagg-3′ and reverse 5′-gttcaaacttctgctcctga-3′ primers and GAPDH forward 5′-tgacatcaagaaggtga-3′ and reverse 5′-tccaccaccctgttgctgta-3′ primers as described previously [13]. To ensure accuracy, an internal reference reaction was performed on the same sample as used for the target gene. The results were standardized with the formula: ΔCT = CT_Ref_ − CT_Target_ and converted to folds of target gene over reference gene (F = 2^- ΔCT^). Data from a minimum of 3 independent experiments was used to quantify gene expression. *P* values of less than 0.05 were considered statistically significant.

### Western blots

Archived PA tumor tissue specimens were requested from the Falk Brain Tumor Tissue Bank, Division of Pediatric Neurosurgery at A&RLCH under IRB protocol #2005-12252. Total protein was extracted from tumor tissue samples with Tissue Extraction Buffer I (Life Technologies, Cat#FNN0071). For Res186 and Res199 WT or drug resistant cell lines total protein was extracted with Novex® NP40 Cell Lysis Buffer (Life Technologies, Cat#FNN0021) following manufacturer’s instructions. Protein concentrations were quantified with the BCA Protein Assay Kit (Thermo Scientific) with Nanodrop 8000 (Thermo Scientific). Equal amounts of cell lysate were resolved by SDS/PAGE and transferred to nitrocellulose membranes (Bio-Rad). Blocking was performed for 60 min with 5% nonfat dry milk in TBST, followed by blotting with primary antibodies overnight at 4 °C. Primary antibodies included: rabbit polyclonal anti-CD133 (ab19898, 1:500), rabbit polyclonal anti-MDR1 (ab129450, 1:500) or β-actin (1:3,000) from Abcam; rabbit polyclonal anti-phosphorylation-serine 473-Akt (S473-Akt) (1:1,000), rabbit polyclonal anti-phosphorylation threonine 308-Akt (T308-Akt) (1:1,000), mouse monoclonal anti-NF-κB/p65 (1:1,000) and rabbit polyclonal phospho-NF-κB/p65 from Cell Signaling Technology; and rabbit polyclonal anti-Akt (1:2,000), and rabbit polyclonal anti-GAPDH (1:2,000) from Santa Cruz Biotechnology. After extensive washing with TBST, membranes were incubated for 1 h at RT with HRP-conjugated donkey anti-rabbit antibody or donkey anti-mouse antibody (Santa Cruz Biotechnology, 1:5,000), and signal was detected with enhanced chemiluminescence substrate (Bio-Rad). The average intensities of each standard protein band were quantified using Photoshop CS5 (Adobe Systems Incorporated) and compared to band intensities of an internal control protein, GAPDH. The results were column-plotted using GraphPad Prism 7 software. *P* values of less than 0.05 were considered statistically significant.

## Results

### CD133 and MDR1 expression increase in recurrent pediatric PAs following chemotherapy

**Fig. 2:**
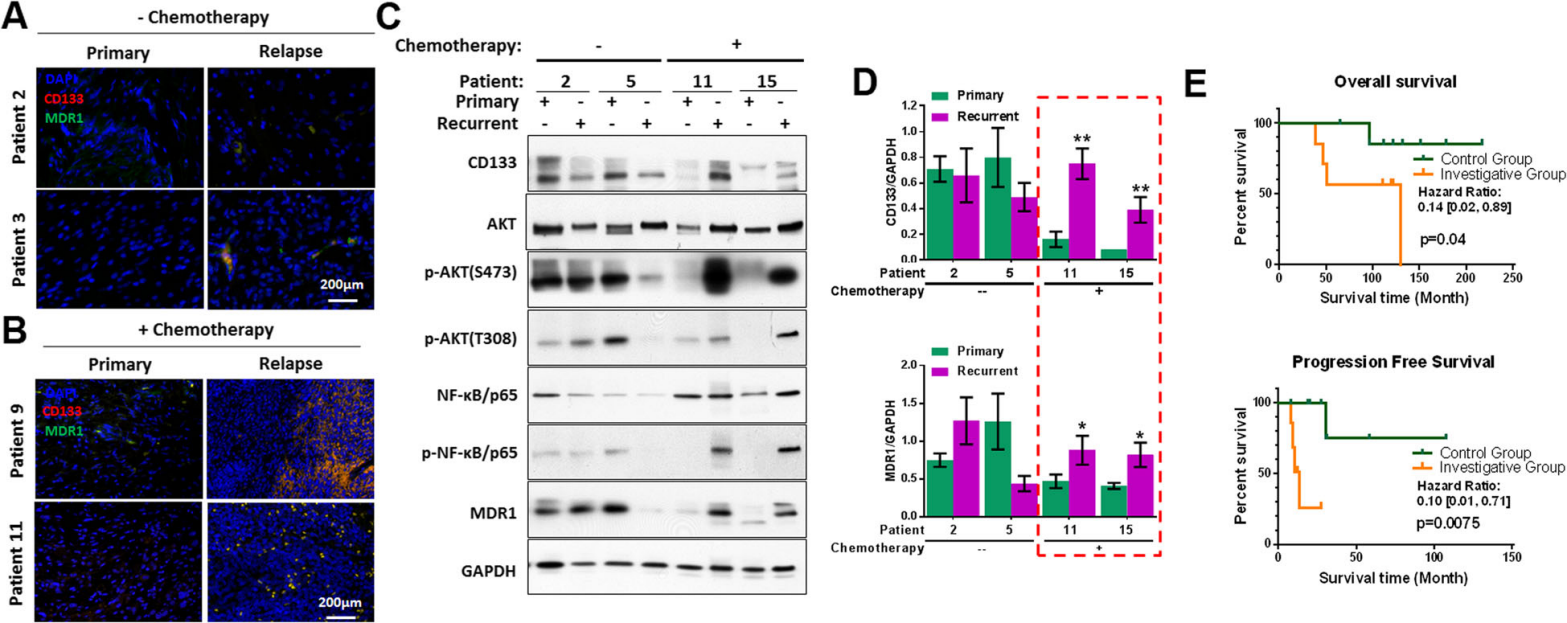
CD133 and MDR1 are present at high levels in recurrent pediatric PAs post chemotherapy. **a** and **b** Representative images show CD133 and MDR1 in primary and matched relapsed PAs without (**a**) and with chemotherapy (**b**). **c** Western blots showing protein expression and activation state for PI3K/AKT/NF-κB signaling mediators in primary and matched relapsed PAs, with and without chemotherapy. **d** Quantification of western blots showing overexpression of CD133 and MDR1 in recurrent PAs with chemotherapy, compared to matched primary tumors. **e** Overall (*top panel*) and progression free (bottom panel) survival of PAs without (control group) or with chemotherapy (investigative group)

CD133 and MDR1 expression levels were examined in paired primary and recurrent pediatric PA tumor tissue. Immunofluorescence with anti-mouse CD133 and anti-rabbit MDR1 antibodies showed CD133 and MDR1 co-expressed in all tumors. Levels of these proteins were similar in paired primary and recurrent tumors from patients who did not receive chemotherapy, whereas protein expression was markedly elevated in recurrent tumors, compared to primary tumor samples, from patients that received chemotherapy (Fig. 2a and b). Western blots confirmed high CD133 and MDR1 expression in recurrent tumors following chemotherapy (Fig. 2c), compared to primary tumors from the same patients. These results indicate an association between CD133 and MDR1 expression, with both increasing in response to chemotherapy.

CD133 regulates MDR1 expression via PI3K/AKT/NF-κB signaling in multidrug resistant adult glioblastoma cells [13]. To investigate this signaling pathway in recurrent pediatric PAs, subsequent to chemotherapy, western blot analysis was performed using protein extracts from archived tumor tissues to detect signaling mediator expression and NF-κB activation. The results show significantly higher protein (CD133/ MDR1), AKT and phosphorylation levels present in recurrent tumors following chemotherapy, relative to primary tumor matched samples (Fig. 2c and d, Additional file 2: Figure S1). Moreover, overall survival (hazard ratio: 0.14, *p* = 0.04) and progression free survival (hazard ratio: 0.10, *p* = 0.0075) of the investigative group who received chemotherapy was lower than the control group without adjuvant treatment (Fig. 2e). These results suggest a role for PI3K/AKT/NF-κB signaling in elevating CD133 and MDR1 expression in pediatric PAs that recur following chemotherapy, similar to previous data in adult drug resistant glioblastoma. Elevated CD133/MDR1 levels most likely contribute to poor patient prognosis.

### Overexpression of MDR1 in drug resistant pediatric PA cells

**Fig. 3:**
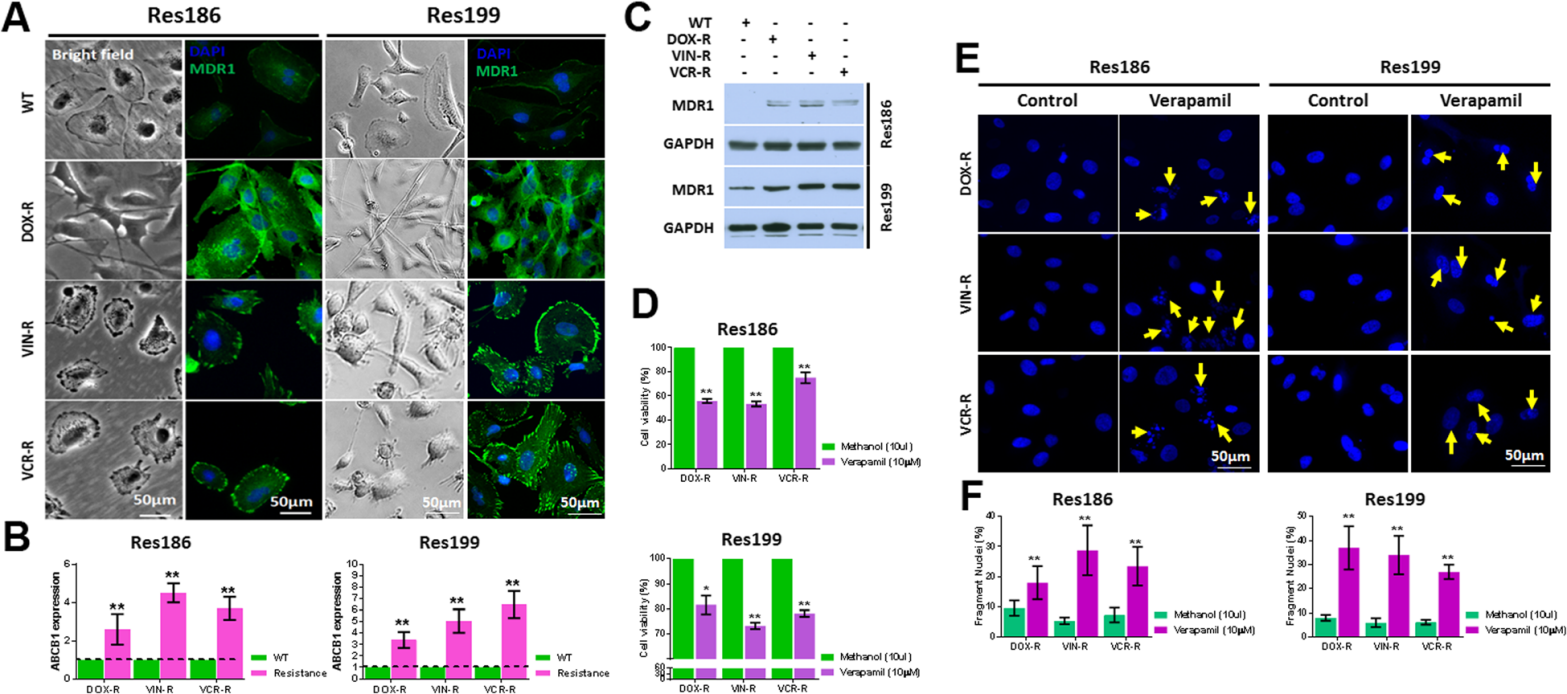
MDR1 is overexpressed and plays a critical role in drug resistant pediatric PA Res186 and Res199 cells. **a** Light micrographs (bright field panels) showing morphology and confocal micrographs (fluorescence panels) showing MDR1 expression with immunofluorescence in drug sensitive (WT), and DOX-R, VIN-R and VCR-R pediatric PA Res186 and Res199 cells. Nuclei are labeled with DAPI (*blue*). **b** and **c** RT-PCR and western blots show ABCB1 (**b**) and its encoded protein MDR1 (**c**) in WT and drug resistant Res186 and Res199 cells. **d** MTS assay showing decreased cell viability of DOX-R, VIN-R, and VCR-R Res186 and Res199 cells in response to 0.1 μg/ml of DOX, VIN or VCR, without (control) or with 10 μM verapamil for 72 h. **e** and **f** Nuclei labeled with DAPI in DOX-R, VIN-R, VCR-R Res186 and Res199 cells treated with 10 μM verapamil for 48 h, or non-verapamil treated controls; fragmentation is indicated by *yellow arrows* (**e**), and quantitated (**f**). (For **b**, **d** and **f**, each column represents the mean ± s.d. of a minimum of three independent experiments done in triplicate; * *p* < 0.05, ** *p* < 0.01)

Pediatric PA Res186 and Res199 cells were used to investigate whether CD133 regulates MDR1 through PI3K/AKT/NF-κB signaling. Res186 and Res199 cells were treated with DOX, VIN and VCR to generate drug resistant cells (DOX-R, VIN-R and VCR-R). Untreated (labeled wild-type (WT)) Res186 and Res 199 cells are relatively drug sensitive. DOX-R, VIN-R and VCR-R cells exhibited morphological changes when viewed under light and confocal microscopes. DOX-R cells were polygonal with long cytoplasmic processes, while VIN-R and VCR-R cells exhibited protrusions along the cytoplasmic membrane (Fig. 3a, Bright field panels). Immunofluorescence (Fig. 3a, fluorescence panels), real-time PCR (Fig. 3b) and western blots (Fig. 3c) showed elevated ABCB1 gene, which encodes MDR1, and higher levels of MDR protein in drug resistant cells relative to WT, respectively.

To determine whether pharmacologic inhibition of MDR1 influences resistant cell response to cytotoxic treatments, DOX-R, VIN-R and VCR-R Res186 and Res199 cells were synchronized overnight in serum-free culture medium, which was then replaced with complete culture medium containing 0.1 μg/ml of DOX, VIN or VCR, with or without 10 μM verapamil, an MDR1 inhibitor [19, 20]. Cell viability and apoptosis were respectively examined with MTS 72 h after treatment and DNA fragmentation analysis 48 h after treatment. Cell viability markedly decreased in drug resistant cells upon co-treatment with chemotherapy plus verapamil (Fig. 3d). Moreover, levels of apoptotic cells, indicated by labeling with DAPI (blue), increased with treatments that included verapamil (Fig. 3e and f). Apoptosis levels were confirmed and quantitated by flow cytometry (Additional file 3: Figure S2A and B). Taken together, our results support increased MDR1 expression in DOX-R, VIN-R and VCR-R Res186 and Res199 cells, relative to WT and MDR1 influence on cell response to chemotherapy.

### CD133 regulates MDR1 expression via the PI3K/AKT/NF-κB signaling

**Fig. 4:**
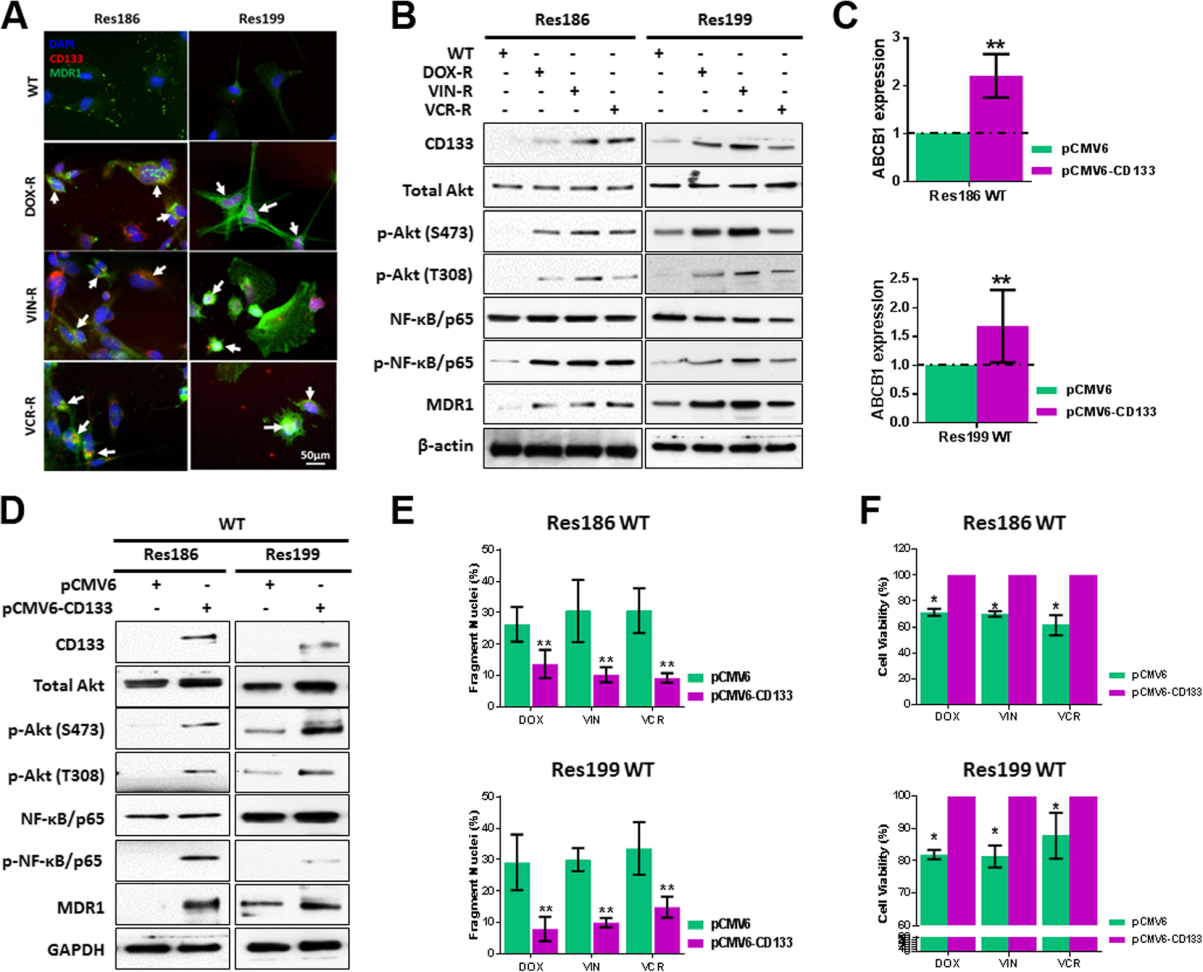
CD133 promotes MDR1 expression through PI3K/AKT/NF-κB signaling in pediatric PA Res186 and Res199 cells and decreases cell chemosensitivity. **a** CD133 and MDR1 co-localize in WT, DOX-R, VIN-R and VCR-R Res186 and Res199 cells (*white arrows*). **b** Western blot results for CD133, total Akt, p-Akt (S473 and T308), NF-κB/p65, p-NF-κB/p65 and MDR1 in WT and Dox-R, VIN-R, VCR-R Res186 and Res199 cells. **c** and **d** Real-time PCR for ABCB1 and western blot results for CD133, total Akt, p-Akt (S473 and T308), NF-κB/p65, p-NF-κB/p65 and MDR1 in WT Res186 and Res199 cells transfected with pCMV6-Myc-DDK (pCNV6) and pCMV6-CD133-Myc-DDK (pCMVCD133). **e** and **f** Nuclear fragmentation and cell viability of WT Res186 and Res199 cells transfected with pCMV6-Myc-DDK (pCNV6) and pCMV6-CD133-Myc-DDK (pCMV6-CD133) in response to 0.01 μg/ml DOX, VIN and VCR treatments for 48 h. (For **c**, **e** and **f**, each column represents the mean ± s.d. of a minimum of three independent experiments done in triplicate; * *p* < 0.01, ** *p* < 0.01)

CD133 expression increases in brain [13, 21, 22] and other tumor cells following chemotherapy [23–25]. Further, CD133 co-localizes with MDR1 after chemotherapy in pediatric medulloblastoma [12] and ependymoma [11]. As shown here, by immunofluorescence, CD133 and MDR1 co-localize and expression levels are elevated in DOX-R, VIN-R and VCR-R Res186 and Res199 PA cells, compared to WT (Fig. 4a).

We have previously shown that CD133 regulates MDR1 expression through PI3K/AKT/NF-κB signaling in adult GBM cells [13]. To determine whether this occurs in drug resistant Res186 and Res199 PA cells, we used western blot analysis. CD133, p-Akt (S473 and T308), phospho-NF-κB/p65, and MDR1 all increased in DOX-R, VIN-R and VCR-R cells, compared to WT cells (Fig. 4b). Transfection of Res186 and Res199 WT cells with pCMV6-CD133, elevated CD133 expression and increased: ABCB1 transcript, which encodes MDR1 (Fig. 4c), p-Akt (S473 and T308), phospho-NF-κB/p65, and MDR1 (Fig. 4d). Consequently CD133 overexpression decreased nuclear fragmentation (Fig. 4e) and increased cell viability (Fig. 4f) of WT Res186 and Res199 cells in response to 0.01 μg/ml DOX, VIN and VCR. Taken together, the present results, along with our previous findings [13] implicate PI3K/AKT/NF-κB signaling as being of central importance to the effect of CD133 on MDR1 expression and hence chemosensitivity.

### Targeting CD133 improves chemotherapeutic efficacy in vitro

**Fig. 5:**
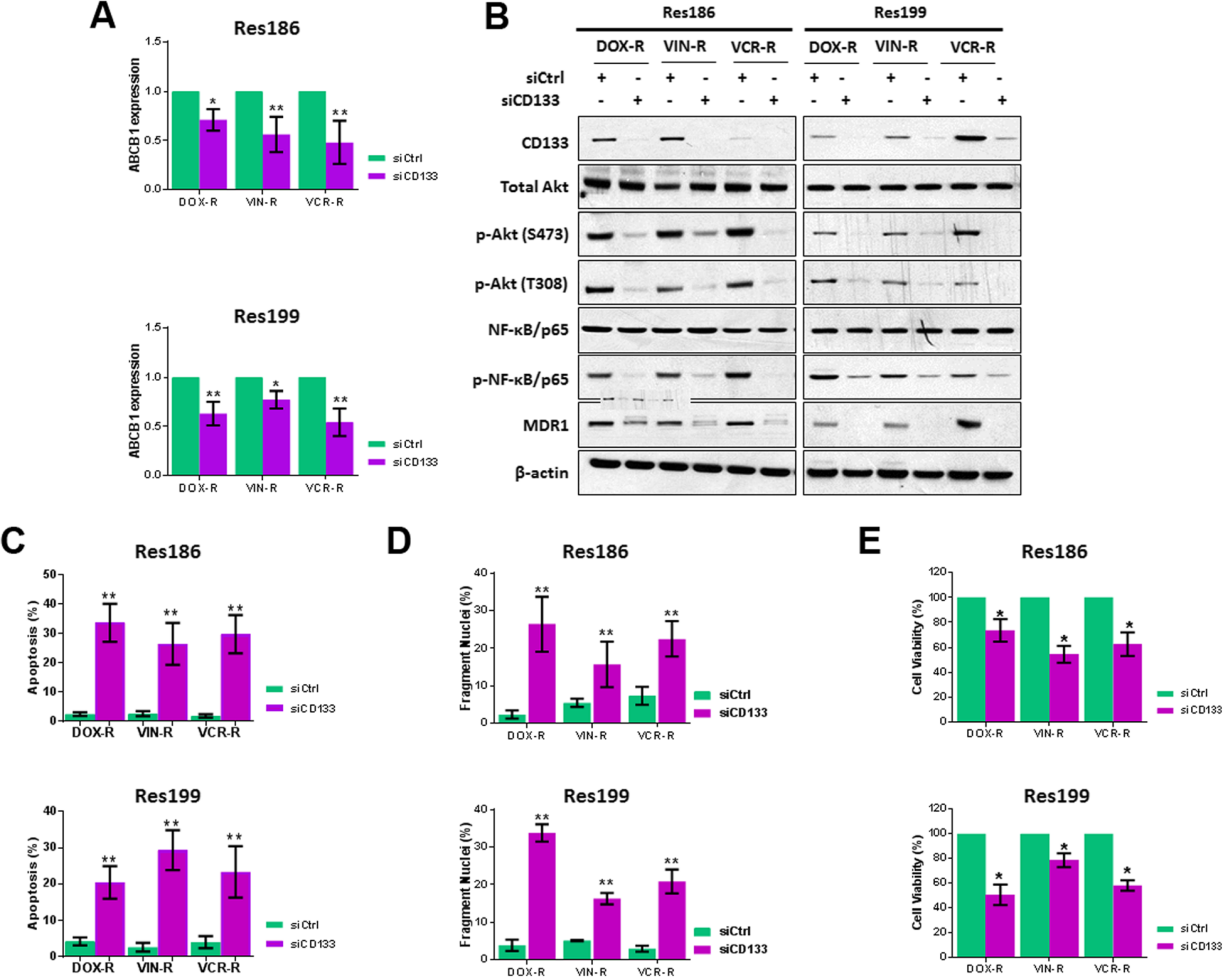
Targeting CD133 improves chemotherapeutic efficacy in drug resistant Res186 and Res199 cells. **a** and **b** Real-time PCR and western blots for ABCB1 gene expression and protein alterations of CD133, total Akt, p-Akt (S473 and T308), NF-κB/p65, p-NF-κB/p65 and MDR1 in DOX-R, VIN-R, VCR-R Res186 and Res199 cells transfected with siCD133, compared to control (siCtrl) cells, respectively. **c**, **d** and **e** Apoptosis with quantitative analysis by flow-cytometry, nuclear fragmentation and cell viability of DOX-R, VIN-R, VCR-R Res186 and Res199 cells transfected with siCD133 or siCtrlfor 48 h, followed by continued treatment with 0.1 μg/ml of indicated drug for 72 h after transfection, respectively. (For **a**, **c**, **d** and **e**, each column represents the mean ± s.d. of a minimum of three independent experiments done in triplicate; * *p* < 0.05, ** *p* < 0.01)

CD133 regulates MDR1 in drug resistant glioblastoma [13] and, as shown here, pediatric PA Res186 and Res199 cells. Given this relationship, down-regulation of CD133 should decrease MDR1 expression and increase tumor cell chemosensitivity. To investigate this, drug resistant cells, DOX-R, VIN-R and VCR-R, cultured in the presence of the drug they had acquired resistance to, were treated with siRNA against CD133. SiRNA treated cells were then examined for ABCB1 gene expression (real-time PCR), protein expression (western blots), apoptotic response (nuclear fragmentation and flow-cytometry) and cell viability (MTS assay). Down-regulation of CD133 in Res186 and Res199 drug resistant cells decreased ABCB1 gene (Fig. 5a) and encoded protein expression (MDR1), and reduced p-Akt (S473 and T308), phospho-NF-κB/p65, and CD133 (Fig. 5b). Furthermore flow-cytometry showed increased numbers of apoptotic cells in drug resistant cells treated with siCD133 (Fig. 5c), and fragmented nuclei increased in these cells, compared to cells treated with control siRNA (Fig. 5d). Finally, cell viability of drug resistant cells significantly decreased following siCD133 treatment (Fig. 5e). In total, our results indicate that suppressing CD133 expression increases chemotherapeutic efficacy of drug resistant PA cells.

## Discussion

CD133, AKT, NF-κB and MDR1 were examined in matched primary and recurrent, with or without chemotherapy, PAs, from pediatric patients. These proteins were present at higher levels in tumors that recurred following chemotherapy (Fig. 2). Increased expression was also observed in PA cell lines, Res186 and Res199, following chemotherapy. MDR1 was regulated by CD133 through PI3K-Akt-NF-κB signaling, consistent with our previous findings in drug resistant adult glioblastoma [13]. These results suggest that a novel adjuvant chemotherapeutic regimen, including agents that inhibit CD133 expression may improve prognosis for children with PAs that are not surgically curable, through MDR1 down-regulation.

MDR1, mediated drug resistance is the most extensively characterized drug resistance mechanism in brain tumors. This ATP-driven transmembrane drug transporter decreases intracellular drug accumulation through decreased drug uptake and increased drug efflux. High ABCB1 gene expression, which encodes MDR1, is associated with chemo-resistance and poor outcome in many types of brain tumors, including medulloblastomas [26, 27], gliomas [28], ependymomas [29–31] and PAs [32]. However, the underlying basis for increased MDR1, in response to chemotherapy, is poorly understood. Chemotherapeutic drugs are known to induce changes within histone proteins that are associated with MDR1 promoter sequences, these changes include increased histone 3 (H3) acetylation [33] and induction of methylated H3 lysine (K) 4 [34], both of which enhance multidrug resistance. Protein kinase C, RAS, p53, and MDM2 are known to modulate MDR1 expression and phosphorylation [35].

CD133, a cell surface marker for neural stem cells, is present in malignant brain tumor tissues. Transplantation of CD133 positive tumor cells, but not CD133 negative cells, into NOD/SCID or nude mice produces tumors that are similar to patient tumors from which they were derived [36, 37]. Moreover, CD133 positive tumor cells possess enhanced chemo- and radio-resistance, and contribute to tumor recurrence and progression [38–40].

The purpose of this study was to examine MDR1, CD133, and their potential interaction in pediatric PAs, using both patient samples and PA cell lines Res186 and Res199. Previous studies have shown CD133 positive cells in adult and pediatric low-grade gliomas including pediatric PAs [14, 15] and Res186 cells [16]. In this study, CD133 positive cells were found to be present at low levels in primary and recurrent tumors from patients not receiving chemotherapy. In contrast, CD133 levels were substantially elevated in relapsed tumors from PA patients that received chemotherapy. MDR1 expression levels in patient PAs were paralleled by CD133 levels. Elevation of CD133 expression in recurrent tumors following chemotherapy may be due to selection of a pre-existing subpopulation of CD133+ cells, or through induction of CD133 expression, or both. The basis for induced expression is poorly understood, but increasing evidence suggests involvement of tumor microenvironment and epigenetic factors [41–43]. For instance, hypoxia, in response to chemotherapeutic agents, affects CD133 expression [41], and CpG hypomethylation of CD133 promoter sequences, as well as methylation of histone H3 K4 and K27 residues, known to upregulate CD133 [42–44].

Two PA cell lines Res186 and Res199 were used to investigate whether CD133 has a regulatory role in MDR1 overexpression. Drug resistant Res186 and Res199 cell lines were developed by extended treatment with DOX, VIN or VCR. As in observed in recurrent pediatric tumors treated with chemotherapy, CD133 positive cells increased in drug resistant cells. Furthermore, drug-resistant cells showed elevated MDR1 expression. Suppressing CD133 expression in these cells decreased MDR1. Western blot results suggest that CD133 may impact MDR1 levels through PI3K-Akt-NF-κB signaling. The findings in this study, in combination with other reports, support the possibility that CD133 positive cells are involved in tumorigenesis and recurrence in pediatric PAs.

## Conclusions

In conclusion the results presented support a critical role for CD133 in chemotherapy, not only in malignant brain tumors, as suggested previously, but also in low-grade gliomas including pediatric PAs. Future studies should focus on the development of a two-pronged chemotherapeutic approach, targeting CD133 and MDR1 as a means to eradicate CD133 positive drug resistant cells and ultimately improve treatment outcomes for patients with brain tumors, including recurrent PAs.

## Additional files

Additional file 1: Clinic characteristics of 143 pediatric PAs patients from 1994 to 2012 were reviewed from the database at the Division of Pediatric Neurosurgery at Ann & Robert H. Lurie Children’s Hospital (A&RLCH), Northwestern University Feinberg School of Medicine. (XLSX 23 kb)
Additional file 2: Figure S1. Quantification of western blot results showing overexpression of pAKT(S473, T308) and pNF-ƙB/p65 in recurrent PAs with chemotherapy, compared to matched primary tumors. (TIF 52 kb)
Additional file 3: Figure S2. Inhibition of MDR1 induces cell apoptosis in drug resistant Res186 and Res199 cells. A) Flow cytometry profiles for DOX-R, VIN-R, VCR-R Res186 and Res199 cells, either untreated or treated with 10 μM verapamil for 72 h. B) Number of apoptotic cells based on flow results. (For B, each column represents the mean ± s.d. of a minimum of three independent experiments done in triplicate. ** *p* < 0.01). (TIF 89 kb)

## Abbreviations

CNS: Central nervous system
DOX: Doxorubicin
DOX-R: Doxorubicin-resistant
FFPE: Formalin fixed paraffin embedded
IRB: Institutional review board
MDR1: Multidrug resistant protein 1
MRP: Multidrug protein
MTS: 3-(4, 5-dimethylthiazol-2-yl)-5-(3 carboxymethoxyphenyl)-2-(4-sulfophenyl)-2H-tetrazolium
PA: Pilocytic astrocytoma
P-gp: P-glycoprotein
PI: Propidium iodide
VCR: Vincristine
VCR-R: Vincristine-resistant
VIN: Vinblastine
VIN-R: Vinblastine-resistant
