# Supplementary figures and images for: Targeting CD133 improves chemotherapeutic efficacy of recurrent pediatric pilocytic astrocytoma following prolonged chemotherapy
Source: Mol Cancer. 2017 Jan 31;16:21. doi: 10.1186/s12943-017-0593-z (PMC5282778; doi:10.1186/s12943-017-0593-z)

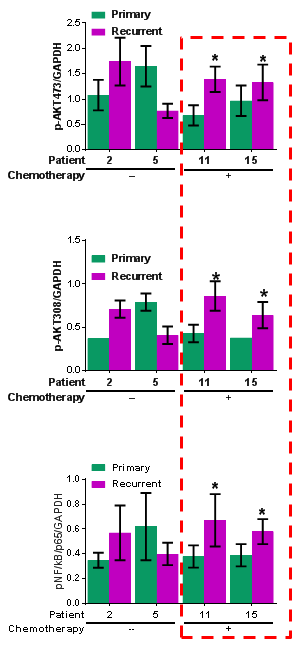

Supplement: Additional file 2: Figure S1. — Quantification of western blot results showing overexpression of pAKT(S473, T308) and pNF-ƙB/p65 in recurrent PAs with chemotherapy, compared to matched primary tumors. (TIF 52 kb) [file 12943_2017_593_MOESM2_ESM.tif]

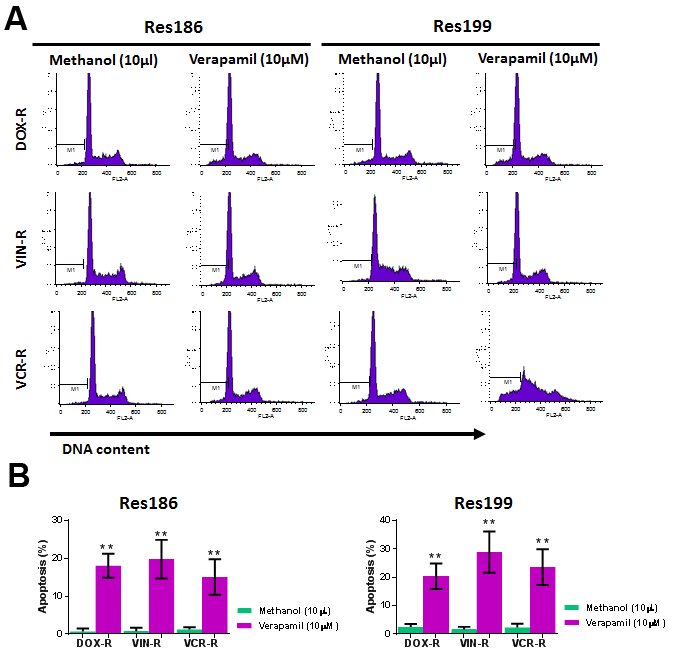

Supplement: Additional file 3: Figure S2. — Inhibition of MDR1 induces cell apoptosis in drug resistant Res186 and Res199 cells. A) Flow cytometry profiles for DOX-R, VIN-R, VCR-R Res186 and Res199 cells, either untreated or treated with 10 μM verapamil for 72 h. B) Number of apoptotic cells based on flow results. (For B, each column represents the mean ± s.d. of a minimum of three independent experiments done in triplicate. ** p < 0.01). (TIF 89 kb) [file 12943_2017_593_MOESM3_ESM.tif]
